# Operative management versus non-operative management of rib fractures in flail chest injuries: a systematic review

**DOI:** 10.1007/s00068-016-0721-2

**Published:** 2016-08-29

**Authors:** Jaap Schuurmans, J. C. Goslings, T. Schepers

**Affiliations:** Trauma Unit, Department of Surgery, Academic Medical Center, Meibergdreef 9, PO Box 22660, 1100 DD Amsterdam, The Netherlands

**Keywords:** Flail chest, Surgery, Systematic review, Randomized clinical trials, Mortality, Pneumonia

## Abstract

**Purpose:**

Flail chest is a life-threatening complication of severe chest trauma with a mortality rate of up to 15 %. The standard non-operative management has high comorbidities with pneumonia and often leads to extended Intensive Care Unit (ICU) stay, due to insufficient respiratory function and complications. The aim of this literature study was to investigate how operative management improves patient care for adults with flail chest.

**Methods:**

Randomized-controlled trials comparing operative management versus non-operative management of flail chest were included in this systematic review and meta-analysis. PubMed, Trip Database, and Google Scholar were used for study identification. We compared operative-to-non-operative management in adult flail chest patients. Mean difference and risk ratio for mortality, pneumonia rate, duration of mechanical ventilation, duration of ICU stay, duration of hospital stay, tracheostomy rate, and treatment costs were calculated by pooling these publication results.

**Results:**

Three randomized-controlled trials were included in this systematic review. In total, there were 61 patients receiving operative management compared to 62 patients in the non-operative management group. A positive effect of surgical rib fracture fixation was observed for pneumonia rate [ES 0.5, 95 % CI (0.3, 0.7)], duration of mechanical ventilation (DMV) [ES −6.5 days 95 % CI (−11.9, −1.2)], duration of ICU stay [ES −5.2 days 95 % CI (−6.2, −4.2)], duration of hospital stay (DHS) [ES −11.4 days 95 % CI (−12.4, −10.4)], tracheostomy rate (TRCH) [ES 0.4, 95 % CI (0.2, 0.7)], and treatment costs (saving $9.968,00–14.443,00 per patient). No significant difference was noted in mortality rate [ES 0.6, 95 % CI (0.1, 2.4)] between the two treatment strategies.

**Conclusions:**

Despite the relatively small number of patients included, different methodologies and differences in presentation of outcomes, operative management of flail chest seems to be a promising treatment strategy that improves patients’ outcomes in various ways. However, the effect on mortality rate remains inconclusive. Therefore, research should continue to explore operative management as a viable method for flail chest injuries.

## Introduction

Flail chest (FC) is a life-threatening complication of severe chest trauma and occurs in up to 15 % of chest wall injuries [1]. “Flail chest occurs when three or more adjacent ribs are fractured in at least two places, creating a chest wall segment that moves paradoxically from the chest wall [2].” The paradoxical movements make breathing less efficient, resulting in poor oxygenation of blood and potential asphyxia [3]. The trauma that results in a flail chest can also lead to lung contusion and pneumothorax, which worsens blood oxygenation [4–6]. Among flail chest patients, 40 percent get pneumonia making it the most common non-acute complication [7]. Consequently, flail chest is a dangerous chest trauma complication with a relatively high chance of causing asphyxia or death.

The standard hospitalization practices of FC management currently include (non) invasive ventilation and pain control. Although mechanical ventilation management has improved throughout the years, FC is still associated with long Intensive Care Unit (ICU) stays, a high morbidity rate, and high treatment costs [8]. It is crucial to find the best treatment strategy to optimize FC management, to benefit patient care, and to continue researching the benefits of operative management.

Operative management has gained increasing attention for flail chest settings. Even though there are more publications on operative management, the number of randomized-controlled trials is limited (RCT). Recently published systematic reviews, therefore, combine prospective and retrospective publications for meta-analysis. Most of these studies and reviews suggest operative fixation of flail chest could be an alternative treatment to reduce duration of ICU stay, days on mechanical ventilation (DMV), mortality rate, and treatment costs [6, 9–11]. A systematic review of randomized-controlled trials only has not been published to date. The aim of this literary study was to assess whether operative management of FC has a positive effect on patient outcomes compared to current treatment methods.

## Methods

PubMed, Trip Database, and Google Scholar were used for study identification. Titles and abstracts of all hits were screened for relevance before being included. The last search was performed in November 2015.

### PubMed search

[[[“Rib Fractures”(Mesh)] OR “Flail Chest”(Mesh) OR rib fracture*(tiab) OR rib fractures*(tiab) OR flail chest*(tiab)] AND [“General Surgery”(Mesh)] OR “Surgical Procedures, Operative”(Mesh) OR surgery*(tiab) OR operative*(tiab) OR operation*(tiab) OR surgery (subheading)].

### Trip database search

This database was searched combining the terms ‘flail chest’ and ‘operation’ or ‘surgery’.

### Google scholar

Google scholar was searched using the following search terms; ‘surgical management flail chest randomized controlled trials’ and ‘randomized controlled trials comparing flail chest management.’

### Study selection

A total of 2096 publications were screened to determine relevance for this systematic review. Only RCTs comparing operative management in adults with flail chest with non-operative management were included in this meta-analysis. Publications had to be written in English to be included. Three publications on RCTs satisfied these criteria (Table 1). No RCTs were excluded due to the use of a language other than English. The reference list of these three included publications was screened to identify any additional studies, but none were conclusive. We also looked at the reference list of recent systematic reviews by Leinicke et al. and Slobogean et al., but no other RCTs were found.

**Table 1:** General characteristics of the included randomized clinical trials comparing operative and non-operative managements of flail chest

Author	Year	Location	Operative management *N*=	Non-operative management *N*=	Outcomes reported	Operative strategy	Quality
Tanaka et al. [6]	2002	Japan	18	19	Pneumonia, DMV, ICU stay, TRCH, FVC	7,8 inion plates and bicortical screws	Moderate
Granetzny et al. [13]	2005	Egypt	20	20	Mortality, DMV, ICU stay, DHS, FVC, FEV_1_	Kirschner wires, stainless steel wire or both	Moderate
Marasco et al. [5]	2013	Australia	23	23	Mortality, pneumonia, DMV, ICU stay, DHS, TRCH, FVC, FEV_1_	Metal hooks and Judet’s struts	High

*n* number of patients, *DMV* duration of mechanical ventilation, *ICU stay* intensive care unit stay, *TRCH* tracheostomy rate, *FVC* forced vital capacity, *DHS* duration of hospital stay, *FEV*_*1*_ forced expiratory volume

### Data extraction and quality assessment

Quality assessment of the RCTs was done using the Cochrane Library Checklist for Randomized-Controlled Trials [12]. Publication scoring 0–2, 3–4, or >5 were considered low, moderate, and high qualities, respectively. Primary outcomes for this meta-analysis were mortality and pneumonia rate. We, therefore, collected data on these outcomes. In addition, we investigated clinical data consisting duration of mechanical ventilation, duration of ICU stay, pulmonary infection rate, days in hospital, incidence of lung contusion, rate of tracheostomy, and management costs. We also collected general data, such as: demographics (age and gender), smoking rate, number of patients, injury severity score (ISS), and operative strategy. All different operative approaches in the publications included were pooled. The included RCTs provided no information regarding method of blinding.

If additional information was required for meta-analysis, the authors of the included RCTs were requested to provide this information. Meta-analysis and statistic significance testing were performed using Review Manager 5.3.5 (The Cochrane Collaboration). A random-effect model, instead of fixed-effects, was used for meta-analysis if *I* ^2^ was larger than 75 % (Fig. 3). Differences with a *P* value under 0.05 were considered significant.

## Results

**Fig. 1:**
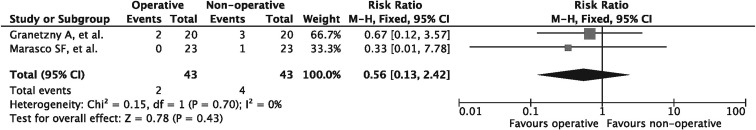
Risk ratio for mortality in patients with flail chest treated with operative managements versus non-operative management. *SD* standard deviation, *M–H* Mantel–Haenszel Method

**Fig. 2:**
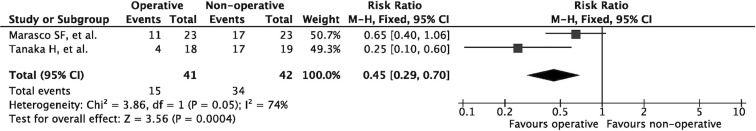
Risk ratio for pneumonia in patients with flail chest treated with operative managements versus non-operative management. *SD* standard deviation, *M–H* Mantel–Haenszel Method

In total, there were 61 patients receiving operative management compared to 62 patients in the non-operative management group (Table 1). Primary outcomes for this meta-analysis were mortality and pneumonia rate. No significant difference in risk ratio (RR) for mortality between the operative and non-operative management groups was seen [ES 0.6, 95 % CI (0.1, 2.4)] (Fig. 1) [5, 13]. Incidence of pneumonia was with an RR of 0.5 significantly lower in the operative management group [ES 0.5, 95 % CI (0.3, 0.7)] (Fig. 2).

**Fig. 3:**
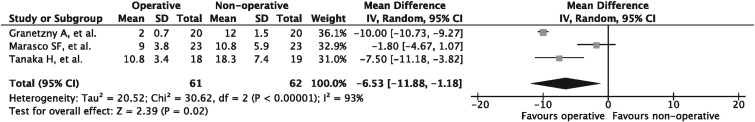
Difference in duration (days) of mechanical ventilation between operative and non-operative managements in patients with flail chest. *SD* standard deviation, *IV* inverse variance

**Fig. 4:**
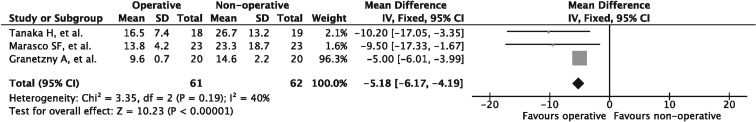
Difference in duration (days) of Intensive Care Unit stay between operative and non-operative managements in patients with flail chest. *SD* standard deviation, *IV* inverse variance

**Fig. 5:**
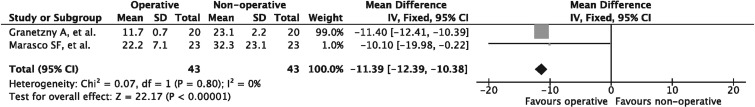
Difference in duration (days) of hospital stay between operative and non-operative managements in patients with flail chest. *SD* standard deviation, *IV* inverse variance

Other significant favorable results for operative management were: duration of mechanical ventilation [ES −6.5 days 95 % CI (−11.9, −1.2) *P* = 0.0006] (Fig. 3) [5, 6, 13]; duration of ICU stay [ES −5.2 days 95 % CI (−6.2, −4.2) *P* > 0.00001] (Fig. 4) [5, 6, 13]; days in hospital [ES −11.4 days 95 % CI (−12.4, −10.4) *P* < 0.0001] (Fig. 5) [5, 13]; tracheostomy rate [ES 0.4, 95 % CI (0.2, 0.7)] [5, 6], and forced vital capacity [ES 6.1 %, 95 % CI (2.7, 9.5)] [5, 13]. Although Tanaka et al. do not describe the exact differences in FVC, they too state that the operative management group had a significantly better FVC after 3 months compared to non-operative management [6]. Forced Expiratory Volume in one second (FEV_1_) did not differ between management groups [ES −0.8 95 % CI (−4.2, 2.7)] [5, 13].

There were more chest wall deformities, such as stove-in chest, reported in the non-operative management group by Granetzny et al. at 45 % for non-operative compared to 5 % of patients in the operative management groups. [13] Tanaka et al. also suggest that non-operative management is not always successful in preventing chest wall deformities and thereby supports the results described in the publication by Granetzny et al. [6]. When looking at management costs, operative management appeared to be $10.000 to $14.443 less expensive according to Tanaka et al. and Marasco et al., respectively [5, 6].

No significant difference in returning to work ratio after 12 months was found by Tanaka et al., ratios being 16/18 for the operative management group and 12/19 for the non-operative management group [6]. Operative management did have a positive effect on returning to high-intensity jobs though with ratios of 3/18 and 13/19 for the non-operative and operative management group, respectively (*P* < 0.05) [6].

When comparing the quality of life of the management groups, no significant difference was noted between the two management groups by Marasco et al. when using the Short Form-36 Quality of Life questionnaire [5].

## Discussion

This meta-analysis shows that operative management of flail chest improves the outcome of patients concerning pneumonia, DMV, ICU stay, days in hospital, tracheostomy rate, FVC, and treatment costs. FVC results by Tanaka et al. could, although significantly in favor of operative management, not be pooled. FVC results by Tanaka et al. were, therefore, compared without meta-analyses to see if they supported or weakened results found by the other publications included.

There were some differences between the publications included, which might have influenced the outcome of this meta-analysis. For instance, patients included by Granetzny et al. were, with an average age of 38.3 years, youngest of all three publications, and in general, younger patients have higher survival rates in trauma events [14]. Another discrepancy between the studies is the injury severity score (ISS) with the lowest average ISS (17.4) in the study by Granetzny, compared to the ISSs reported by Tanaka et al. (ISS = 31.5) and Marasco et al. (ISS = 32.5). Higher ISSs correlate with more severe trauma cases and higher mortality rates [14]. There were also differences in statistic analysis with very small standard deviations (SD) in the study of Granetzny. However, after excluding data reported by Granetzny et al. from meta-analysis, outcomes are still significantly in favor of operative management.

The reported complications were different between the included studies, which might be due to discrepancies in the definition of complications. Pneumonia is an important complication in flail chest settings with an average incidence of 40 % [7]. Granetzny et al. conducted a comprehensive report of all complications in the operative management group, but they did not include pneumonia, whereas two of their fatal cases were due to pneumonia [13].

Tanaka et al. investigated patients’ quality of life between operative and non-operative managements of flail chest. However, the study did not report the number of patients excluded due to incapability to complete questionnaires. In addition, the publication by Tanaka et al. did not report on any fatalities, which is exceptional with an average ISS between 30 and 35 [6].

The included RCTs used different randomization strategies to limit the risk of bias. Granetzny et al. used random numbers balanced with blocks of ten patients. The publication does not provide information regarding concealment of allocation. Tanaka et al. used a randomization chart for randomization. Researchers were not blinded, but treatment was protocol driven to limit the risk of bias. Marasco et al. used a computer generated code using block randomization with block size of four for their randomization. After randomization, an opaque envelope was opened with the treatment assignment. The treating intensivists were not blinded, but ventilatory support and placement of tracheostomy were protocol driven.

A comparable systematic review was published in July 2015 by Cataneo et al. [18]. This systematic review included the same RCTs that were included in our study. However, we managed to pool additional data on duration of mechanical ventilation and duration of ICU stay (Figs. 3, 4, respectively). These analyses could not be done using the data described in the RCTs, so we requested the authors of the RCTs by Granetzny et al. and Marasco et al. to provide original data. We received the additional data from Prof. Dr. A. Boseila and Prof. Dr. S. Marasco. This enabled us to pool data regarding DMV and ICU stay. Besides these analyses, we also compared treatment costs for both strategies. Therefore, our study provides additional insights in the differences between operative and conservative managements of flail chest.

A systematic review and meta-analysis, comparing both retrospective and prospective studies, were published in 2013 by Leinicke et al. [9]. A positive effect on DMV (4.5 days), ICU stay (3.4 days), and days in hospital (3.8 days) was seen in favor of operative management, which is in concordance with our results [9]. In addition, Leinicke et al. found a positive effect of surgery on pneumonia and tracheostomy rate with relative risks being 0.45 and 0.25, respectively [9]. We too found favorable results for operative management when looking at these outcomes. The only difference between data sets was the mortality rate, since Leinicke et al. found a significantly lower mortality rate in the operative management group [9]. The most recent publication about FC management by Xu et al. found a shorter duration of mechanical ventilation and ICU stay when using operative management, supporting our findings [2]. Pneumonia rate was also significantly lower in their operative management group, but tracheostomy rate did not differ between both groups in the publication by Xu et al. [2].

A retrospective study comparing operative-to-non-operative management of multiple rib fractures was published in October 2015 [15]. Patients treated with surgical rib fracture fixation were matched two-to-one to non-operative patients with similar injuries. There were some differences between both treatment groups, which made it difficult to compare both treatment groups. For example, flail chest was present in 79 % of patients in the operative management group compared to 23 % of patients in the non-operative management group. A subgroup analysis was made, leaving out patients with any degree of head injury. The only significant differences were DMV (3 to 5 days) and tracheostomy rate (5–23 %), for operative and non-operative management, respectively. There were no significant differences in treatment costs.

Several studies have been published on the effect of different pain management strategies. Baker et al., for example, published a retrospective observational study in which they investigated the use of analgesics in management of patients with primary thoracic injuries [16]. This study, however, could not identify an analgesic mode that lowered the chance of pulmonary complications. Carrier et al. published a systematic review in 2009 [17]. RCTs comparing epidural analgesia to other analgesic modes in adults with traumatic rib fractures were included in their meta-analysis. No significant difference was found in mortality rate, ICU stay, hospital stay, or DMV. DMV did significantly decrease when only studies using thoracic epidural analgesia with local anesthetics were pooled. The RCTs included in our meta-analysis provided little information on pain control. Tanaka et al. mentioned that they used continuous epidural anesthesia prior to randomization for pain control, but no specific data were reported. Marasco et al. reported that they chose not to assess pain in their study, because they thought that it would be confounded by other injuries than the flail chest.

Although only randomized-controlled trials were used for this meta-analysis to reduce the risk of inclusion bias, there are several limitations due to inconsistencies throughout the studies. For example, no information regarding method of blinding participants and researchers could be found. However, it is hard to avoid bias when comparing operative management to conservative strategies. The studies also differed in research compilation, specifically with regard to treatment protocols and operative strategies. In addition, the number of RCTs and sample sizes was relatively small. Therefore, more RCTs should be performed, comparing not only operative versus non-operative management, but also expand on viable operative strategies to improve treatment. Since the number of patients suffering from a flail chest is limited, national or international multicenter trials should be initiated to improve the reliability of the outcome. Research should also be continued to explore the benefits of different operative strategies.

## Conclusions

Despite the limitations of the small number of RCTs published, the number of patients included that, diversity in operative strategy and differences in outcome presentation, this meta-analysis of randomized-controlled trials showed several positive effects of operative management compared to non-operative management in flail chest settings. Primary outcomes for this analysis were mortality rate and incidence of pneumonia. The operative management group showed a significant lower incidence of pneumonia, whereas mortality rate did not differ between treatment groups. Operative management of flail chest might, therefore, be a promising treatment strategy that could not only improve patient’s outcome, but also lower treatment costs. These observations might be of value for surgeons treating patients with flail chest.
